# A sclerochronology defined 600-year baseline of marine dynamics in the North Sea

**DOI:** 10.1098/rstb.2024.0036

**Published:** 2025-07-10

**Authors:** David Reynolds, Evgeny Genelt-Yanovskiy, Anna Genelt-Yanovskaya, Emma Northey, Paul Butler, Tamara Trofimova, Martina Conti, Kirsty Penkman, Qian Huang, Bernd Schöne, James Scourse

**Affiliations:** ^1^ Department of Earth and Environmental Sciences, University of Exeter, Penryn Campus, Penryn TR10 9FE, UK; ^2^ Department of Chemistry, University of York, York YO10 5DD, UK; ^3^ Institute of Geosciences, University of Mainz, 55128 Mainz, Germany

**Keywords:** sclerochronology, *Arctica islandica*, climate, North Sea, environmental baseline

## Abstract

*Arctica islandica* is a long-lived marine bivalve mollusc that inhabits the continental shelf seas of the North Atlantic Ocean at subpolar and temperate latitudes. Growth increments formed within *A. islandica* shells can be analysed to form annually resolved and absolutely dated sclerochronologies that are considered the marine counterpart to dendrochronologies. Here, we present an updated *A. islandica* chronology from the Fladen Ground (North Sea) that spans between CE 1410 and 2021. The *A. islandica* chronology significantly (*p *< 0.05) covaries with observational local sea surface productivity, subsurface seawater temperature and sea surface salinity. These analyses highlight the potential sensitivity of the chronology to the variability in the regional seawater temperature and salinity, and atmospheric circulation patterns (wind stress and the summer North Atlantic Oscillation). The connection between the atmospheric circulation patterns and *A. islandica* shell growth is likely due to fluctuations in the strength of the Atlantic inflows into the North Sea and local mixing. This highlights the significant potential in using *A. islandica* records to provide long-term baselines of North Sea marine dynamics. These baselines are critical for understanding the impact future changes in regional oceanic and atmospheric variability may have on North Sea ecosystems and fisheries.

This article is part of the theme issue ‘Shifting seas: understanding deep-time human impacts on marine ecosystems’.

## 1. Introduction

**Figure 1.**
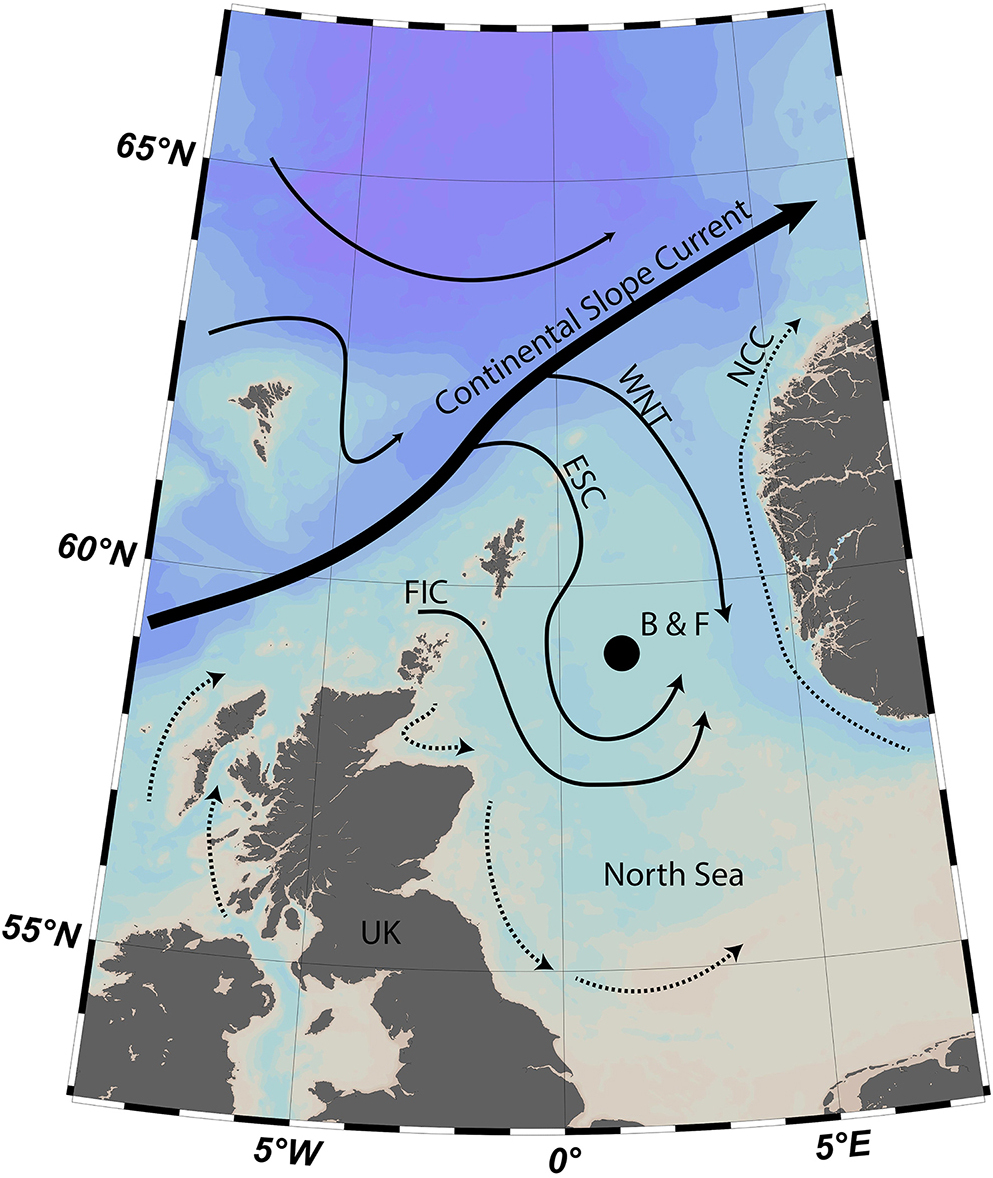
Map showing the location of stations B and F in the Fladen Ground (North Sea). Arrows show the approximate position of sea surface currents. Solid black arrows show major inflows from the North Atlantic Ocean from the Continental Slope Current via the East Shetland Current (ESC), the western flank of the Norwegian Trench (WNT), and the Fair Isle Current (FIC), which also provide pathways for the influx of Atlantic water into the North Sea. The Norwegian Coastal Current (NCC) is also shown.

The North Sea is a shallow (typically <100 m) shelf sea situated between the British Isles and continental Europe (figure 1). The region sustains commercially important fisheries that have increased dramatically since the onset of industrialization in the 1700s [1]. The resulting increase in fishing pressure has driven significant shifts in the abundance and size of fish in the North Sea [2]. However, in addition to modern fishing pressures, variability in the size and distribution of North Sea fish has been associated with the interplay between physical climate and ecosystem dynamics [3]. For example, analyses have highlighted that the distribution of the commercially important Atlantic cod (*Gadus morhua*) within the North Sea has shifted to the north and deepened, likely in response to the warming surface waters [4]. In addition, a significant decline has been identified in North Sea primary productivity over recent decades [5]. This decline, attributed to the increase in sea surface temperature and reduction in riverine input, is argued to be acting as a bottom-up forcing contributing to recruitment and productivity shifts in higher trophic level species [5]. However, despite North Atlantic waters significantly influencing the climate of the northern North Sea, the shifts within North Sea primary production and zooplankton abundance differ from those of the adjacent northeast Atlantic Ocean [6]. These differences are hypothesized to be due to the shallow and stratified nature of the North Sea increasing local rates of sea surface warming in contrast to the muted warming to cooling that has been observed in the Subpolar Gyre (SPG) region of the North Atlantic [7]. Given the socioeconomic importance of the North Sea region and the observed trends across North Sea ecosystems, there is a pressing need to constrain the uncertainties in our understanding of the climate-ecosystem coupling across this region.

Climate variability in the North Sea and adjacent northwest European region is strongly linked to coupled ocean−atmosphere variability in the North Atlantic [8]. Atlantic waters flow into the North Sea via both the English Channel in the south (0.1 Sv) and via the Fair Isle Current (FIC), the East Shetland Current (ESC) and the Western Norwegian Trench (WNT) in the north (1.4 Sv combined [9]; figure 1). The English Channel water, which makes up a relatively small proportion of the northern North Sea water, is transported along the northwest European coastline towards the Skagerrak [10]. The North Atlantic inflows bring significant nutrient loading and heat that enhances biological productivity across the region [11]. The strength of these inflows is linked to regional ocean and atmospheric circulation patterns associated with the North Atlantic Oscillation (NAO) and SPG strength. The NAO, defined as the sea-level air pressure (SLP) difference between the Iceland low and the Azores (or Gibraltar) high, influences the strength and position of the winds and air masses situated over northwest Europe and the North Sea [12]. NAO-driven variability in wind strength and direction acts as a top-down forcing on surface mixing and Ekman transport in the region. In addition, heat transported into the North Sea is linked to broad-scale North Atlantic circulation variability (e.g. Atlantic Meridional Overturning Circulation (AMOC) and SPG strength [8]). Consequently, given predicted declines in AMOC’s buoyancy forcing, models are predicting a significant decline in the rate of Atlantic inflow into the North Sea over the coming decades [13].

Instrumental records provide invaluable data about the state of modern ecosystems and climate variability in the North Sea spanning recent decades (e.g. [14]). However, variability over the interval these records represent (typically 1970 to present) is heavily influenced by anthropogenic factors (both fishing pressure and anthropogenic climate change). Therefore, the magnitude of anthropogenic influences during this period likely constrains our ability to accurately identify and quantify natural linkages between physical and biological variability across this region. To address this challenge, previous studies have highlighted the potential of the long-lived marine bivalve mollusc *Arctica islandica* to provide high-resolution absolutely dated multi-centennial baselines of marine variability [15–29].

*A. islandica* is a bivalve mollusc that inhabits soft-bottom benthic communities across the temperate and subpolar continental shelf seas in the North Atlantic, and is common in the North Sea [24]. The growth increments and associated geochemical composition (collectively referred to as sclerochronology) of *A. islandica* shells in the North Sea have been extensively studied [24–26,30–37]. These studies have highlighted the potential for building long-term records of past marine variability by exploiting the synchronous nature of growth among individuals growing at the same time [34]. Consequently, through the application of dendrochronologically based statistical techniques (e.g. crossdating), it is possible to build sclerochronological records that extend beyond the lifespan of a single specimen (e.g. [15]). These studies have highlighted that variability in the width of the annually formed growth rings in *A. islandica* is sensitive to a range of environmental parameters including primary production [25,38,39] and water temperature [21,34].

This study seeks to further investigate the links between *A. islandica* growth in the northern North Sea (Fladen Ground; figure 1) and both local and regional climate. The Fladen Ground is ideally suited for examining the influence of Atlantic inflows because of its proximal location to the ESC inflow. Additionally, *A. islandica* is abundant across this region of the North Sea providing sufficient quantities of live and dead (subfossil) material to build absolutely dated multi-centennial chronologies [37]. The aims of this study are to: (i) establish an updated multi-centennial record of *A. islandica* shell growth from the present day back over past centuries; (ii) apply the regional curve standardization (RCS) techniques to facilitate the construction of a new sclerochronology designed to capture marine dynamics across the broadest possible spectrum of frequency domains; and (iii) exploit the new multi-centennial record to evaluate the possible linkages between the northern North Sea climate dynamics and regional climate variability.

## 2. Methods

### (a) Sample collection

This study utilized live- and dead-collected *A. islandica* samples that had previously been collected by the *RV Scotia* in May and June 2001 and by the *RV Prince Madog* in June 2004. These samples have been utilized to construct several absolutely dated sclerochronologies and a corresponding carbon stable isotope record [34,37]. To supplement these earlier collections, additional live and dead *A. islandica* shell material was collected in May 2022 from the same locations previously sampled (station B located at 59° 7' 16.212" N, 0° 10' 0.012" E (WGS84), water depth 125 m; and station F at 59° 23' 0" N, 0° 30' 0" E, water depth 130 m; figure 1). Samples were collected using a 1 m wide mechanical dredge deployed by the *RRS Discovery*. In total, the dredge was deployed eight times at station F and 21 times at station B. The deployments at each site were within a 1 km square centred around the site coordinates. Of the 475 live caught specimens, a total of 98 and 16 were kept from stations B and F, respectively. The remaining live caught specimens were returned to the seafloor. In total, 1160 and 326 empty shell valves (dead specimens) were collected from stations B and F, respectively. All empty shell valves were kept for further processing. Full details of the sampling are available in the cruise report [40]. Previous work has highlighted that *A. islandica* contain a common growth signal over approximately an 80 km range in the Fladen Ground [34]. Given that stations B and F are 40 km apart, the samples at both locations could be utilized to build a single *A. islandica* sclerochronology.

Biometric information (shell length, height, maximum height, width, mass, bioerosion, ligament preservation, margin preservation and bioerosion) was recorded for each live- and dead-collected specimen. Although shell taphonomic condition is not a reliable guide to shell antiquity [36], shell size and condition metrics can provide an indication as to whether a specimen is likely to yield a readable growth record and whether a sample is likely to be of a greater longevity. Therefore, the biometric information was used to select optimal shells (shell length ≥ 65 mm, and with minimal bioerosion) for further processing.

### (b) Rangefinder dating

Amino acid geochronology (AAG) is a dating technique relying on the degradation of amino acids as a function of time from the organism’s death. The intra-crystalline fraction of amino acids, preserved in the aragonitic crystal structure of the inner portion of the outer shell layer, showed reliable dates over the Quaternary, with a resolution of approximately 1–2 ka in the Holocene period [41]. While the dating resolution is broad, it is sufficient to determine whether a specimen is likely to have lived during the time interval that immediately precedes the existing FG chronology and therefore warrants further analysis. AAG was used to provide independent rangefinder dates for a subset of the dead-collected shell material. These rangefinder dates provided initial assessments of the potential antiquity of 160 dead-collected specimens. The AAG rangefinders could then be used to select samples that fall within the target time interval (i.e. the period represented by the existing *A. islandica* chronology and the preceding decades to centuries). Samples were selected for AAG dating based on their size (>65 mm shell length) and taphonomic condition (moderate periostracum erosion). For the AAG dating, a section of shell, normally considered an offcut of the standard sclerochronological process (see §2c), was submitted to the University of York for processing. Full details of the AAG sampling, analysis and results are included in Conti *et al.* [41]. The AAG data were then used to select samples that fell within the target time interval (i.e. the period represented by the existing *A. islandica* chronology and the preceding decades to centuries); this subset of samples was then targeted for further chronological analysis.

### (c) Sclerochronological processing

Selected live- and dead-collected *A. islandica* specimens were processed using standard sclerochronological techniques (e.g. [42]). An initial 1−2 cm wide portion of shell, capturing the axis of maximum growth from the umbone to the ventral margin, was cut using a diamond toothed tile cutting saw. The offcut portions of shell, adjacent to the central section, were set aside for AAG rangefinder dating. The central 1−2 cm section, containing the axis of maximum growth, was embedded in Kleer Set polyester casting resin (Metprep, UK). The embedded shell block was then sectioned along the axis of maximum growth using an ATM Brilliant 221 precision saw. One half of the precision sectioned shell was polished using a Merchentek Forcipol 202 polishing table using a progression from coarse to fine sanding grits (127 to 5 μm grit size) and polished using 3 μm diamond solution. The polished surface was etched using 0.1M HCl for 90 s and then rinsed under tap water. Acetate peel replicas were made by applying 30 μm thick acetate sheets to the etched shell surface covered with a meniscus of acetone. The acetate peel replicas were digitally photographed under 5× and 10× magnification using a Nikon DS-RI2 16-megapixel digital camera coupled to a light transmitting microscope. Photomosaics were constructed using a composite of images capturing the growth rings formed in the central cardinal tooth using the Nikon NIS Advanced Elements software. The widths of the growth rings were manually measured using the Ring Measurer app (https://ringdater.github.io/ringmeasurer/).

### (d) Crossdating and chronology construction

The samples collected live in 2022, which have a known date for the outermost increment (2022), were visually crossdated against each other and the samples previously collected live as part of the Butler *et al*. [34] and Estrella-Martinez *et al.* [37] studies. The visual crossdating was then statistically validated using the RingdateR crossdating application [43]. RingdateR uses lead–lag correlation analysis and running correlation analysis to identify the possible crossdates/crossmatches between either two samples or a sample and an existing chronology. In RingdateR, the raw growth ring measurements were detrended using a range of smoothing splines (7 to 32 years), and the stability of the potential matches evaluated using a 21 year running correlation. Samples were deemed to significantly crossdate if the series were found to significantly correlate (based on conventional correlation coefficients, *p*-values (adjusted using a Bonferroni correction to take account of the total number of leads and lags evaluated), and the associated *t*-value statistics). While there is no set threshold for *t*-values, samples were deemed to crossdate if the *t*-value for the given crossdate was notably greater than for any other possible potential crossdate. The ring width measurements of the dead-collected specimens were compared against other dead-collected specimens and with the existing *A. islandica* chronology using RingdateR. Samples identified to potentially crossdate were then visually inspected to validate the statistical crossdating.

Samples that were deemed to significantly crossdate were used to generate a master sclerochronology. To remove the ontogenetic growth trend contained by the raw ring width series, the RCS detrending approach was applied [44,45]. The RCS detrending approach is preferred over other techniques, such as a negative exponential detrending, because RCS preserves a greater proportion of low-frequency variability without also losing the mid- and high-frequency variability [44]. To generate the RCS-based chronology (referred to hereafter as FG RCS), the raw increment width data from each individual crossdated shell were ontogenetically aligned and an arithmetic mean population growth curve calculated. In some samples, where the clarity of the lines reduced in early ontogeny, the growth increment measurements did not represent the full lifespan of the shell. Estimates of the number of increments omitted were made to facilitate the application of the RCS approach (referred to as the pith offset in dendrochronology [44,45]). To evaluate the impact of ontogenetically misaligning the samples, due to uncertainties in the ‘pith offsets’, the ontogenetic alignment of each increment width series was randomly offset between 0 and 10 increments and a new mean population growth curve calculated. These analyses were repeated 1000 times and the associated mean population growth curves compared (electronic supplementary material, figures S3, S4). Each of the 1000 mean population growth curves were then fitted with a 50 year spline. Different length splines (10, 25, 50 and 100 year) and negative exponential RCS curves were also evaluated (electronic supplementary material, figures S4, S5). The 1000 50 year splines were then used as RCS detrending curves to detrend the crossdated raw growth increment width series. The detrending was performed by means of division to minimize ontogenetic trends in variance. The mean chronology was finally constructed by calculating the biweight robust mean of the detrended growth increment width series using the chron function in the Dendrochronology Program Library for R package (dplR; [46,47]).

### (e) Chronology analysis

The periodicities of variability contained in the chronology were evaluated using the multi-taper method (MTM) spectral and wavelet analyses. The MTM spectral analyses were performed using K-Spectra (v. 3.9). The significance of the identified spectra was evaluated against a red noise background. Wavelet analyses were performed using the WaveletComp package (v. 1.1 [48]) in R. The significance of the periodicities identified in the wavelet analyses was evaluated using the WaveletComp’s auto-regressive modelling approach. This approach is similar to the Ebisuzaki Monte Carlo (EMC) methodology in which surrogate time series are generated based on the auto-regressive characteristics of the input dataset [49]. The wavelet analyses are then performed on these surrogate time series 1000 times and the results compared to the periodicities of variability identified in the FG RCS series.

The FG RCS chronology was compared against the Estrella Martinez *et al*. [37] stable carbon isotope (δ^13^C) derived record of herring recruitment. The δ^13^C analyses, which underpin the herring recruitment reconstruction, were performed on calcium carbonate samples extracted from the 14 shells that comprised the previously published Fladen Ground *A. islandica* chronology. Given that these shells are incorporated in the updated FG RCS chronology, there should be no dating uncertainties associated with these analyses. A comparison of the FG RCS chronology against the herring reconstruction provides two important tests. First, other than during the 20th century, the herring reconstruction is constructed from undetrended data [37]. The δ^13^C data had to be detrended during the 20th century to mitigate the influence of the marine Suess effect that is not driven by biological variability [37]. Consequently, the *A. islandica* δ^13^C data should capture environmental variability across all frequency domains prior to *ca* 1900. If the FG RCS and the δ^13^C-based herring reconstruction share a common environmental forcing (e.g. productivity), the herring reconstruction will provide a test as to what extent the FG RCS chronology represents potential low-frequency environmental variability prior to the 20th century. Second, the Fladen Ground δ^13^C record has been interpreted as an index of herring recruitment, linked through variability in seawater productivity. While there are other factors that also influence *A. islandica* shell δ^13^C (isotopic composition of dissolved inorganic carbon (DIC), productivity, air–sea CO_2_ exchange [37,50–53]) the identification of a significant correlation between the herring reconstruction and FG RCS chronology would support the paradigm that *A. islandica* shell growth is likely responding to changes in seawater productivity over past centuries.

### (f) Environmental analysis

To evaluate the sensitivity of the FG RCS chronology to physical climate and biological variability, the FG RCS record was compared to a suite of oceanographic, atmospheric and biological records. For biological variability, the index of primary production was extracted from the Continuous Plankton Recorder dataset [14] for the 1^o^ × 1^o^ latitude by longitude grid box surrounding the sample collection site. The FG RCS record was compared to the monthly and seasonal mean Continuous Plankton Recorder (CPR) record using linear regression analyses.

For physical marine variability, the FG RCS record was compared to seawater temperature and salinity data from the EN4 dataset gridded product [54]. The EN4 product provides monthly mean seawater temperature and salinity data on 42 depth levels from the surface to the deep ocean on a 1^o^ × 1^o^ latitude by longitude grid. The FG RCS record was compared to mean monthly and seasonal temperature, salinity data from the EN4 product using point correlation analysis and linear regression analysis. Significance testing of the point correlation analyses was performed for each individual grid box using the linear regression model function in R. To mitigate the influence of autocorrelation overamplifying the significance of the correlations in the point correlation analyses, an EMC approach was used to further test the significance of the identified correlations. To perform the EMC analyses, the FG RCS chronology was compared to the EN4 data for grid boxes representing the region identified in the point correlation analysis. The surrogateCorr function from the Astrochron package was then used to perform the EMC analysis [55].

Given that observational studies have highlighted the importance of ocean–atmosphere coupling in driving the Atlantic inflows into the North Sea [8], the FG RCS record was compared to a suite of atmospheric products derived from the 20th century reanalysis product [56]. The FG RCS record was analysed against mean monthly and seasonal zonal and meridional surface wind stress, SLP and precipitation using linear regression and point correlation analysis techniques. These point correlation analyses were performed using the KNMI Climate Explorer Facility. The FG RCS record was also compared with the mean monthly and seasonal NAO.

## 3. Results

### (a) Rangefinder dating

Full results of the rangefinder dating by means of AAG are provided in Conti *et al*. [41]. In summary, of the 160 specimens analysed, the AAG technique identified eight shells that dated to the period from 1 to 2 ka BP, and nine shells that likely date to within the last 1000 years.

### (b) Crossdating and growth ring analysis

**Figure 2.**
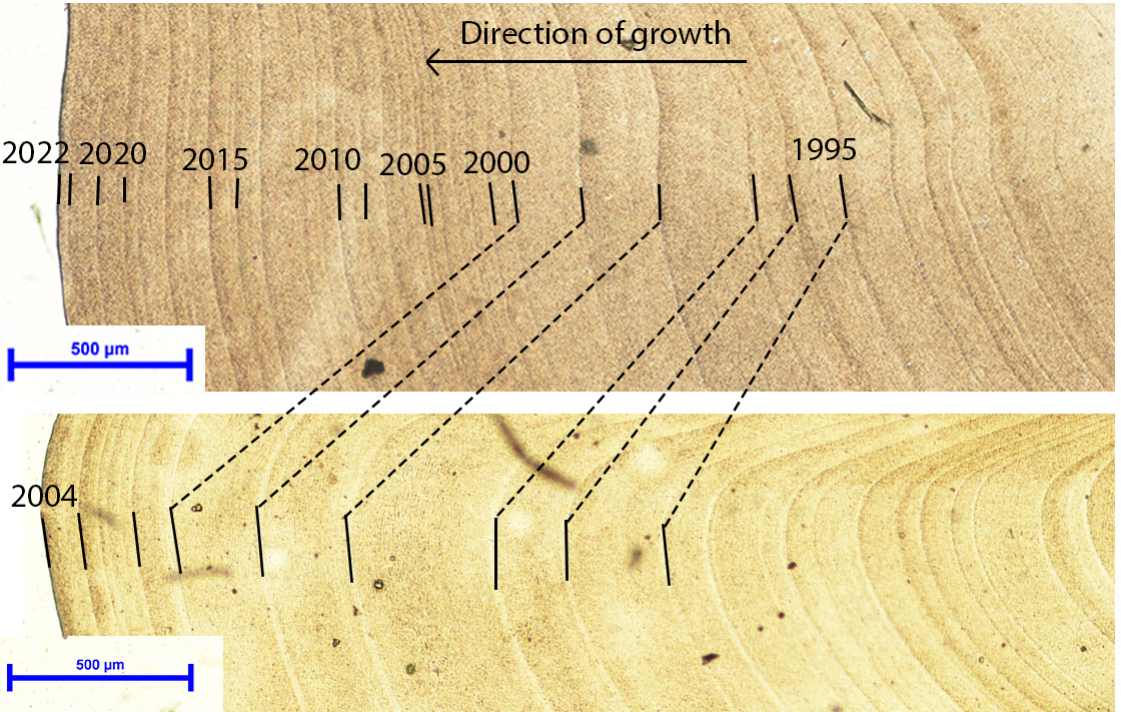
Digital images showing the growth increments in two live-collected *A. islandica* shells collected in 2004 (id no. 0401406L; bottom panel) and 2022 (id no. 22000016; top panel). The dashed black lines highlight the position of signature years across the two live-collected samples. Sample 0401406L was the same sample presented in Butler *et al*. [34]).

In total, out of the 200 specimens analysed, 36 were successfully crossdated to supplement the original 14 shells that comprised the Estrella Martinez *et al*. [37] Fladen Ground chronology (electronic supplementary material, figures S1,S2). The crossdated shells had a mean longevity of 120 ± 36 years, with the longest lived specimen containing 245 increments, and the shortest lived containing 52 increments. The shells collected live in May 2022 were visually and statistically crossdated with the shells that were collected live in 2004, suggesting the most recent counted increment dated to 2022 (figure 2). Therefore, these live-collected samples provide an extension to the previous chronology from 2004 up to 2021. Of the dead-collected specimens that crossdated, a significant proportion crossdated into the existing chronology prior to 1755. The addition of these dead-collected shells added significant replication during the earliest period of the published chronology and, ultimately, extended the chronology back to 1410. Despite successfully crossdating dead shells into the early portion of the chronology, replication during the period from 1729 to 1748 remains low (three shells).

**Figure 3.**
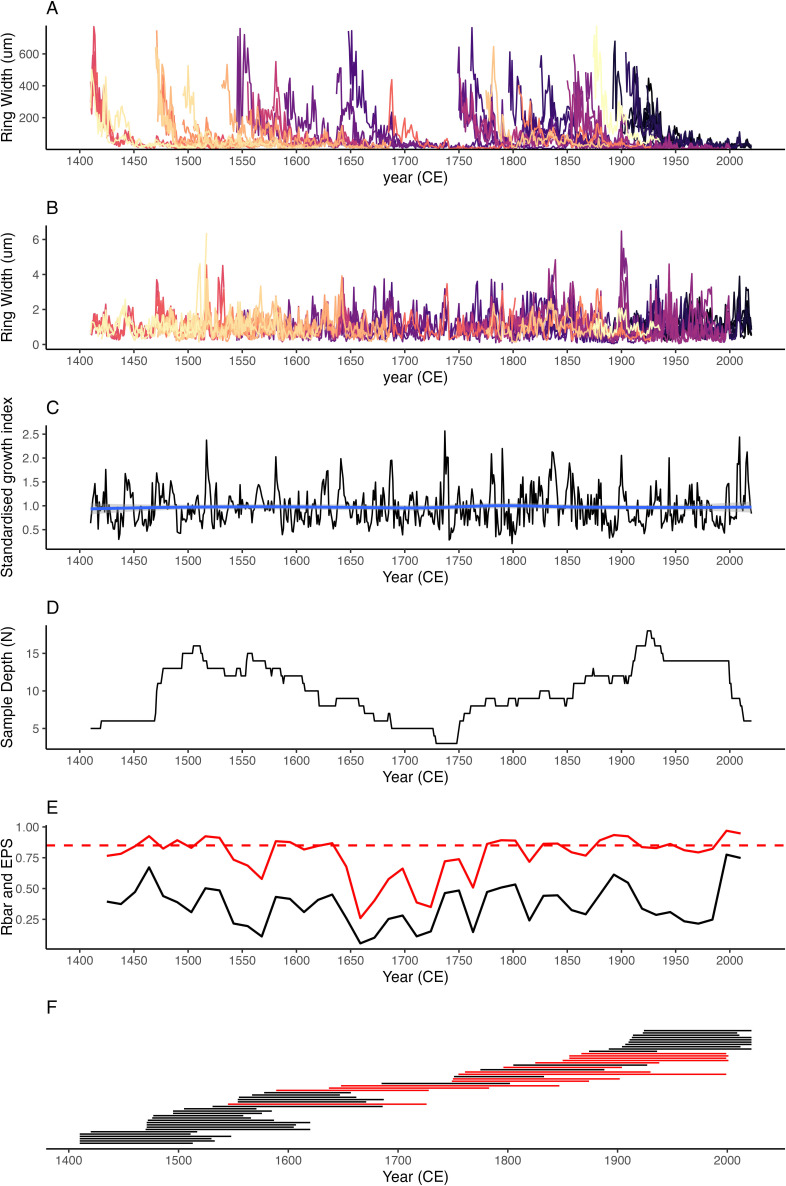
(A) The raw growth increment width series for each shell contained in the FG RCS chronology. (B) The 21 year spline detrended growth increment width data. (C) The biweight robust mean chronology constructed using the 21 year spline detrended data. (D) Plot showing the sample depth (number of shells) representing each individual year contained in the chronology. (E) Running expressed population signal (EPS; red line) and mean correlation between samples (Rbar; black line) calculated using a 100 year running window with 50% overlap. The arbitrary EPS threshold of 0.85 is highlighted with the dashed red line. (F) Plot highlighting the temporal distribution of individual shells, shown as separate red and black horizontal lines, in the revised FG RCS chronology. Shells included in the original record are highlighted in red, with shells incorporated as part of this study shown in black.

Analysis of the individual crossdated shell increment width series against the chronology, constructed excluding the sample being analysed, highlighted that successfully crossdated shells attained mean correlation coefficients of *R* = 0.529 ± 0.117, a mean overlap of 122 ± 34 years and a mean *t*-value statistic of 7.09 ± 2.64. The resulting chronology contained a mean expressed population signal (EPS) of 0.85 over the full chronology, matching the arbitrary EPS threshold of 0.85 [57,58]. The running EPS statistics for the chronology (calculated using a 100 year running window with 50% overlap) highlights that although the mean EPS for the full chronology is 0.85, the EPS falls below the 0.85 threshold between 1650 and 1750. During this period, the FG RCS chronology contains reduced sample depth (figure 3).

### (c) Chronology construction

Evaluation of the influence of ontogenetically misaligning the raw growth increment width data during the construction of the RCS detrending curve highlighted that these offsets have a negligible impact on the resulting RCS curve (electronic supplementary material, figures S3,S4). While the overall uncertainty is small, the analyses do highlight that the uncertainties are heightened during the earliest years of growth. While the overall sample depth is likely sufficient for the application of RCS, the number of samples that represent each ontogenetic age declines with increasing ontogenetic age (notably so >100 years of age). Consequently, it is necessary to apply a generalized RCS curve that provides a fit through the data to mitigate the influence of trends within individual shells in these longer lived samples. The analysis of standard approaches to fitting a generalized curve to the ontogenetically aligned data (e.g. negative exponential and fitted splines) highlighted that, while the choice of curve can influence the amplitude of uncertainties, the choice of curve has little impact on the resulting chronology (electronic supplementary material, figures S4,S5).

**Figure 4.**
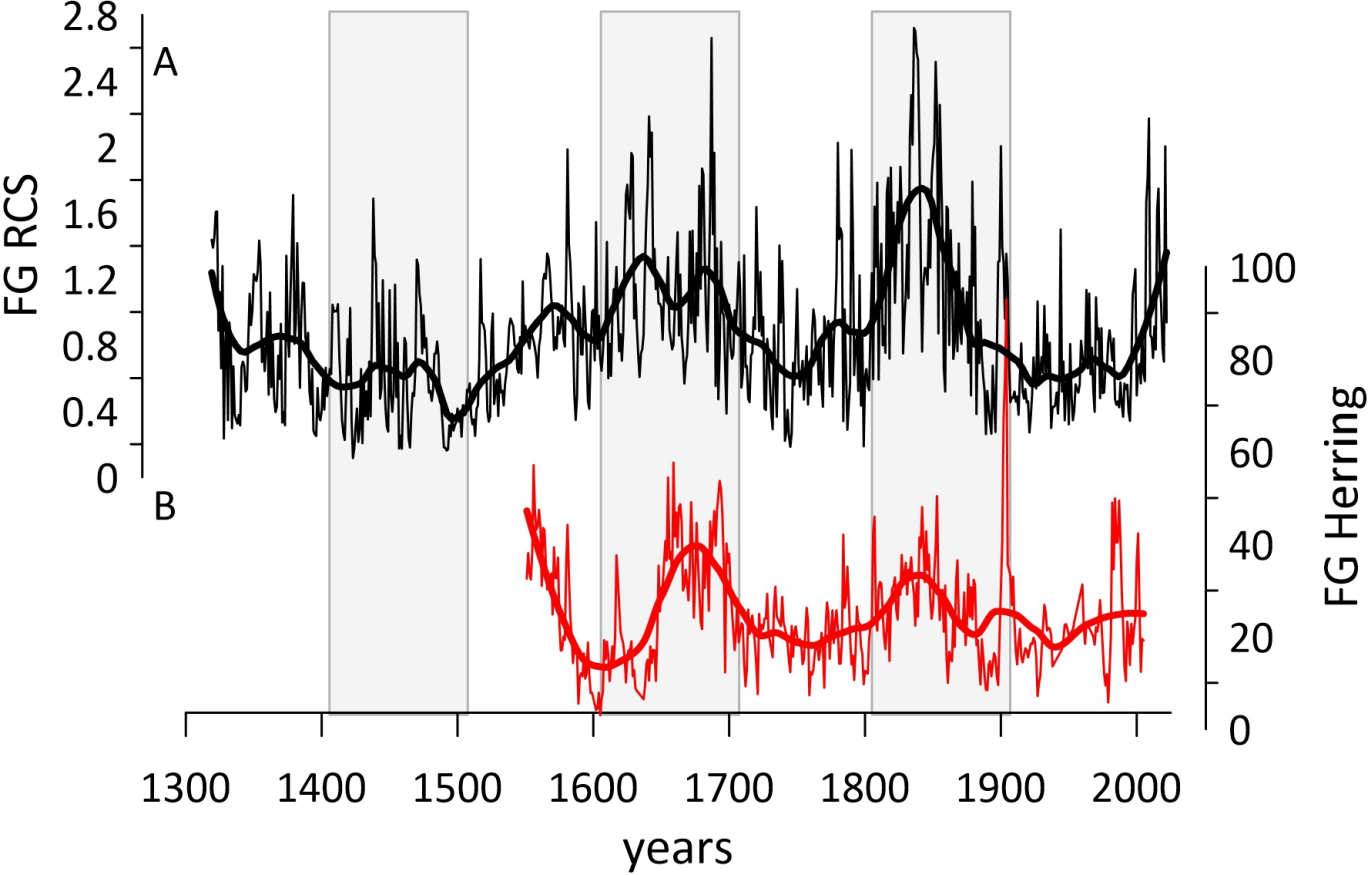
(A) The FG RCS chronology (black line) plotted with a fitted loess first-order low-pass filter (thick black line). (B) The FG *A. islandica *δ^13^C-derived herring reconstruction published by Estrella Martinez *et al.* [37] (red line) and fitted with a loess first-order low-pass filter (thick red line). Shaded vertical bars highlight alternating centuries.

**Figure 5.**
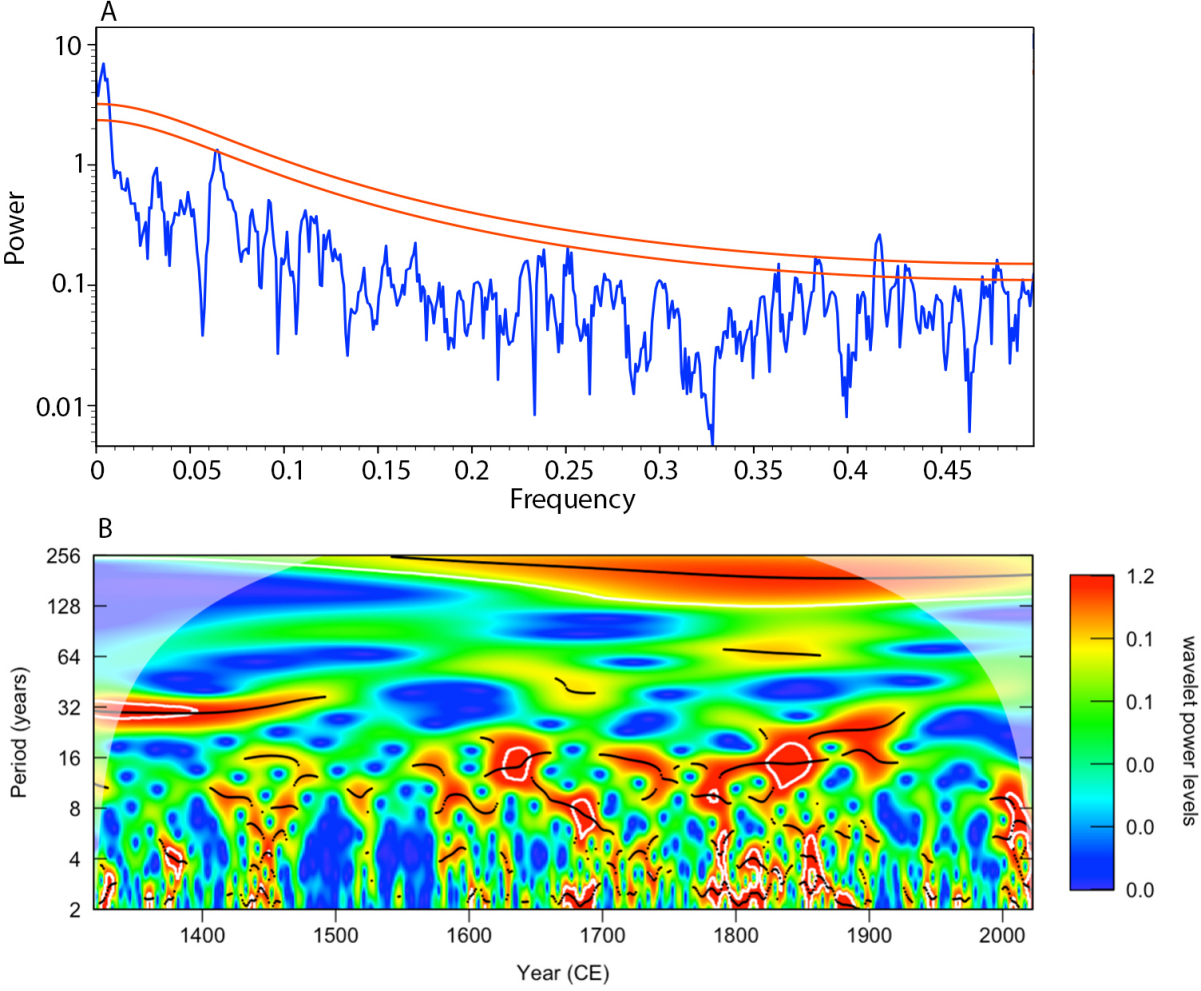
Plots showing the results of (A) the MTM spectral analysis and (B) the wavelet analysis. (A) The blue line shows the power of the variability associated with the frequency bands represented within the FG RCS chronology. The two red lines highlight the 95% (lower line) and 99% (upper line) significance thresholds. (B) Output of the wavelet analysis of the FG RCS chronology. White contours define regions of significant variability (*p* < 0.05). The black lines denote the centre point of the identified periodicity of variability.

The new FG RCS chronology (figure 4) contains notable variability across a range of periodicities over the recorded period (1410−2021). The spectral and wavelet analyses highlight that the FG RCS chronology contains a significant (*p* < 0.01) multi-centennial periodicity variability (figure 5) that appears as periods with enhanced shell growth between 1550−1700, 1825−1875 and 2000−2022. The FG RCS chronology contains periods of reduced shell growth in the decades around 1500, 1750 and during the 20th century. The wavelet analysis also highlights that the FG RCS chronology contains significant decadal scale variability ranging from 8 to 32 years (*p* < 0.05; figure 5B). The analysis suggests a shift may have occurred in the periodicity of decadal variability contained in the FG RCS chronology at approximately 1500. Prior to 1500, the FG RCS chronology contained significant variability at the 32 year periodicity. However, after 1500, the 32 year periodicity is not significant. After 1500, the wavelet analysis suggests the FG RCS chronology contains periodic variability in the 8 to 16 year timeframe. However, while the result is significant (*p* < 0.05), the analysis suggests the significance of the periodicity is not stable.

Comparison of the FG RCS chronology against the *A. islandica* δ^13^C-derived herring recruitment reconstruction highlights that the two series contain significant, albeit weak, covariability during the period of overlap between the two series (*R* = 0.29, *p *< 0.05; significance tested using EMC approach with 1000 simulations). Notably, the herring recruitment reconstruction shows strong centennial scale variability, with peaks in herring recruitment in approximately 1650–1700 and again in the mid-1800s. These periods are synchronous with periods of enhanced shell growth in the FG RCS chronology (figure 4).

### (d) Environmental analysis

**Figure 6.**
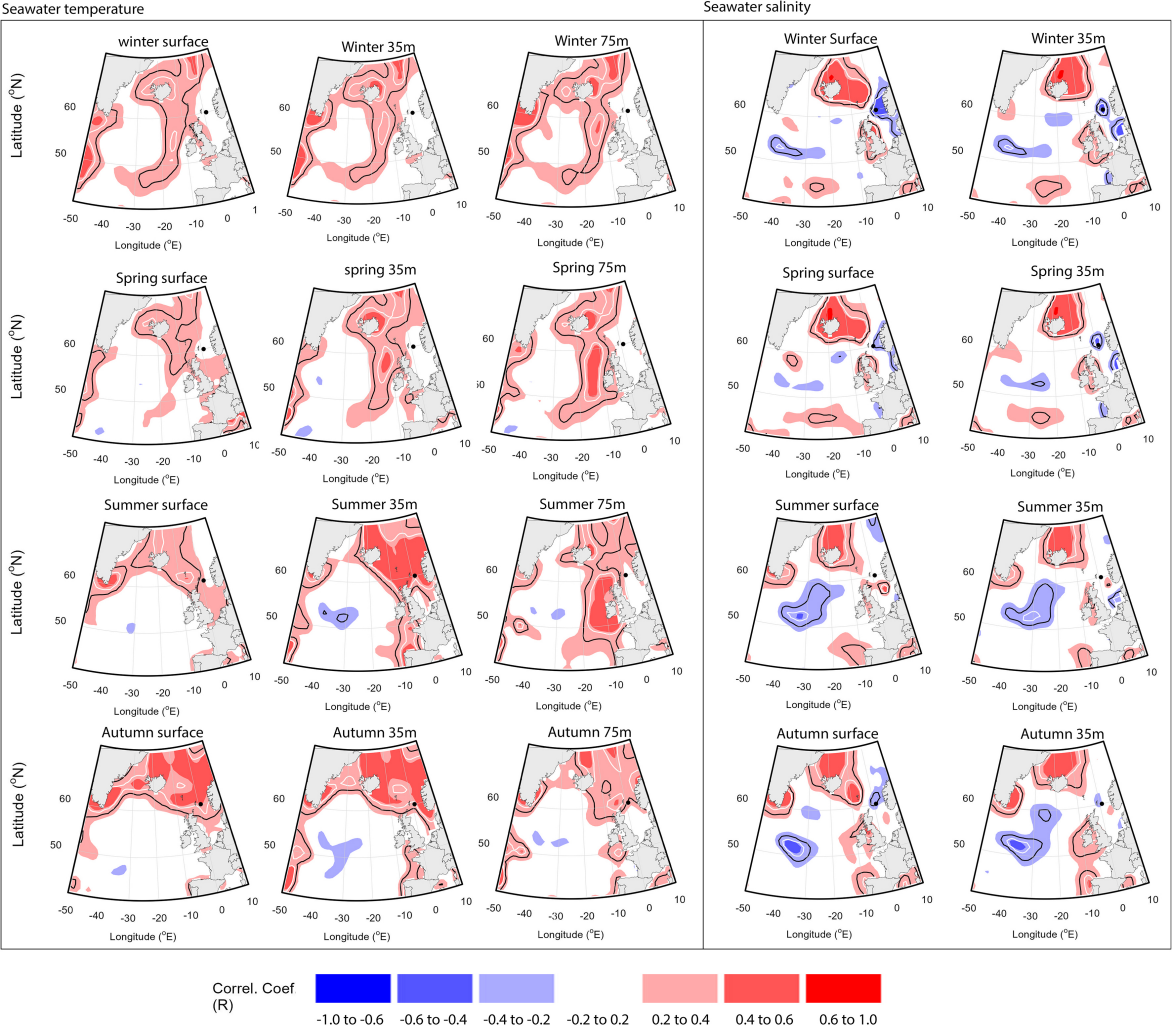
Heat maps showing Pearson correlation coefficients derived from point correlation analyses performed between the FG RCS chronology and mean seasonal EN4 seawater temperature (left panel) and seawater salinity (right panel) at the surface, 35 and 75 m water depths. The black dot marks the sampling location in the Fladen Ground. The black and white contour lines denote regions with significant correlations at the 0.05 and 0.01 significance levels, respectively.

Point correlation analysis identified that the FG RCS chronology significantly correlates with seawater temperatures both locally and in key regions of the North Atlantic region (figure 6). The point correlation analyses highlight that the FG RCS chronology correlates significantly with local mean summer seawater temperature at 35 m water depth, and mean autumn seawater temperatures over the period from 1970 to 2018. The point correlation analyses also highlight a broader pattern of significant positive correlations between the FG RCS chronology and wider North Atlantic seawater temperatures. These correlations broadly follow a horseshoe pattern associated with the boundary of the SPG, with peak correlations occurring west of Ireland and northwestern Scotland. These correlations are present from winter through to the summer months with the strongest correlations occurring at depths between 75 and 300 m during the summer (figure 6).

The point correlation analysis highlights significant negative correlations between the FG RCS chronology and mean sea surface salinity during winter and spring at the sampling location (*p* < 0.01; figure 6). The analyses also highlight significant positive correlations with seawater salinity in the North Atlantic surface waters between the Faroe Isles and around Iceland (*p* < 0.01).

**Figure 7.**
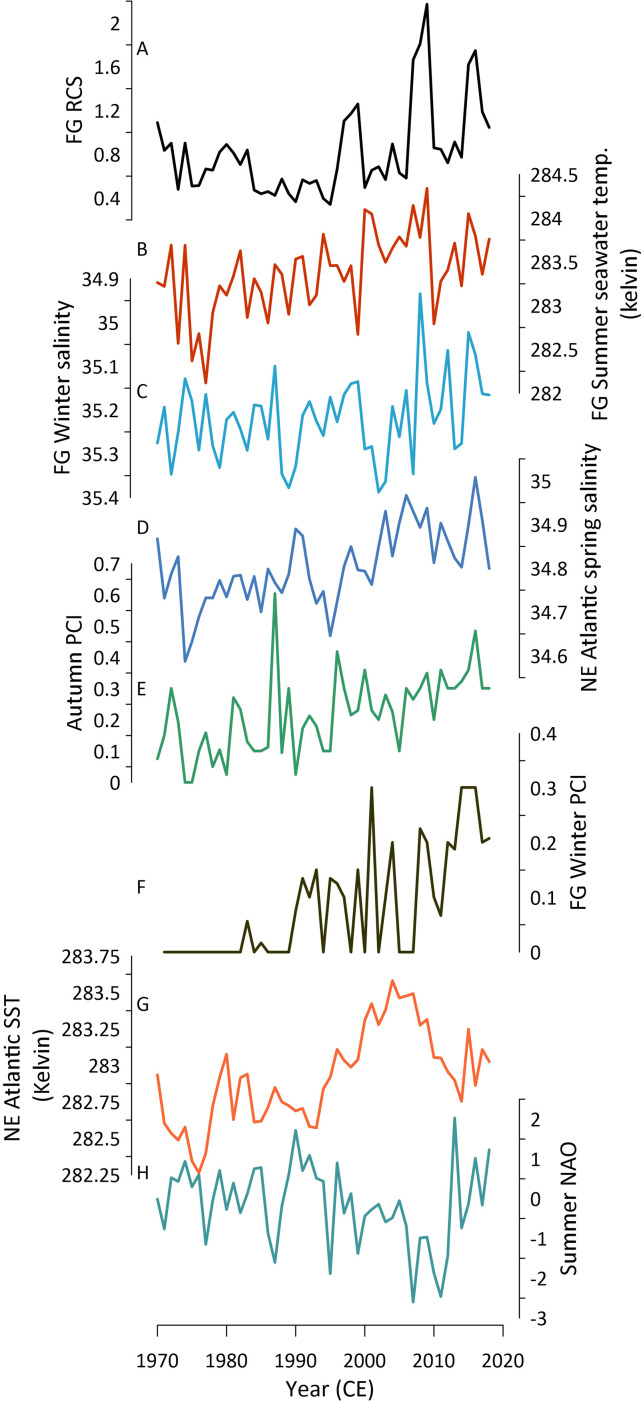
(A) The FG RCS chronology. (B) Mean summer seawater temperatures at 35 m at the Fladen Ground sampling location. (C) Mean winter salinity (0−45 m water depth) at the Fladen Ground sampling location. (D) Mean NE Atlantic seawater spring salinity (25−35 m water depth derived from a 2^o^ grid box centred around 10^o^W, 65^o^N). (E) and (F) Mean autumn and winter surface primary productivity, respectively, at the Fladen Ground sampling location. (G) Mean NE Atlantic seawater temperatures (100−300 m water depth; derived from a 2^o^ grid box centred around 15^o^W, 55^o^N). (H) Mean summer North Atlantic Oscillation. Instrumental data used in plots B, C, D and G are derived from the EN4 gridded product. Data in plots E and F are derived from the Continuous Plankton Recorder dataset. Data in panel H are derived from the NCAR dataset. Locations of data utilized for panels D and G are derived based on the positions of significant correlations identified in figure 6.

Autocorrelation present within the climate time series, together with the substantial number of statistical analyses performed, can lead to the false identification of significant results. To mitigate this issue, the EMC methodology was used to test the likely significance of the identified correlations. The results of the EMC analyses show that the FG RCS chronology significantly correlates with mean summer seawater temperatures at 35 m water depth at the sampling location (*R* = 0.416, *p* < 0.05; figure 7; electronic supplementary material, table S1). The EMC analyses highlight that, although the correlation identified between the FG RCS chronology and mean summer northeast Atlantic seawater temperatures is strong, it is likely not significant when autocorrelation is considered (*R* = 0.454, *p* = 0.1; figure 7; electronic supplementary material, table S2).

The application of the EMC approach to further evaluate the significance of these results suggests that both the correlation with local salinity (*R* = −0.458, *p* < 0.05) and with salinity in the wider northeast Atlantic (*R* = 0.457, *p* < 0.05) are likely significant (figure 7; electronic supplementary material, tables S2 and S3).

**Figure 8.**
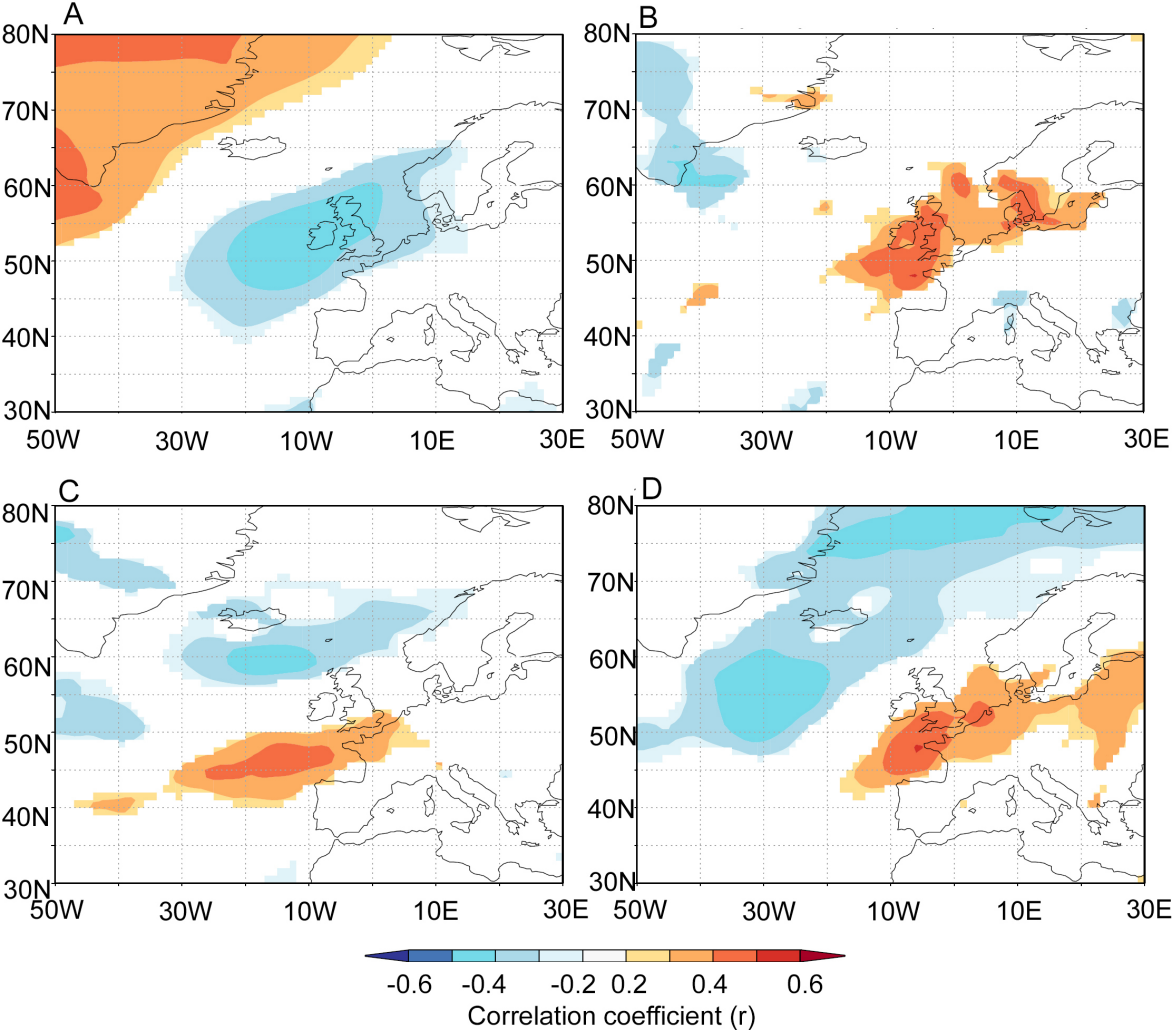
Heat maps showing Pearson correlation coefficients derived using point correlation analysis performed between the FG RCS chronology and mean July to August (A) sea-level pressure, (B) precipitation, (C) zonal wind stress and (D) meridional wind stress. The atmospheric data are derived from the 20th century reanalysis product. The analysis was performed using data between 1970 and 2015. Correlations shown contain *p-*values < 0.1. Positive (negative) correlations with meridional wind stress indicate enhanced shell growth during periods of increased southerly (northerly) winds. Positive (negative) correlations with zonal wind stress indicate enhanced shell growth during periods of increased westerly (easterly) winds.

The point correlation analyses performed between the FG RCS chronology and atmospheric variability highlight significant correlations that follow a bipolar pattern over the wider North Atlantic region. These correlations include a significant positive correlation between zonal and meridional wind stress centred across the UK and in the North Atlantic to the southwest of the UK (figure 8). The analyses also highlight a significant negative correlation with the zonal and meridional wind stress over the central SPG south of Iceland and Greenland. Point correlations highlighted significant correlations between the FG RCS chronology and mean summer SLP that follow a bipolar pattern with negative correlations over the UK, and positive correlations over Greenland (electronic supplementary material figure S7).

Results of the multiple linear regression analyses highlight that the combined influence of the local mean summer seawater temperature (at 35 m), local winter salinity (0−45m) and mean winter and autumn productivity could explain 34% of the variability in the FG RCS chronology (adjusted *R*^2^ = 0.344, *p* < 0.001). Replication of this model with the additional inclusion of mean summer NAO explains 35% of the variance in the FG RCS chronology (adjusted *R*^2^ = 0.350, *p* < 0.0001). Finally, a multiple linear regression model, including the local mean summer seawater temperature (at 35 m), local winter salinity (0−45m), summer NAO, mean winter and autumn productivity and wider NE Atlantic summer seawater temperatures (100−300m) and spring salinity (25−35m), explains 39% of the variance in the FG RCS chronology (adjusted *R*^2^ = 0.388, *p* < 0.001).

## 4. Discussion

Visual and statistical crossdating successfully identified live- and dead-collected specimens, collected during the 2022 sampling, to provide an extension to the previously published Fladen Ground *A. islandica* chronology [37]. The addition of live-collected samples from the 2022 collection extends the previously published record from 2004 up to 2021. The extension of the chronology over this modern interval extends the interval of the overlap between the FG RCS chronology and modern instrumental data. The longer period of overlap provides a greater time frame for performing statistical analyses between the FG RCS chronology and environmental time series. This update is required to facilitate a more robust evaluation of the potential links between the Fladen Ground *A. islandica* growth and North Sea climate dynamics. The crossdating between the live-collected samples from 2022 and the previously published chronology suggests the most recent increment counted is 2022. Given the date of collection (May), it is likely that the 2022 increment was not yet fully formed and, therefore, likely does not represent the full annual growth for that calendar year. Thus, this increment is not included in the final FG RCS chronology.

The successful crossdating of additional dead-collected shells adds significant replication to the previously published *A. islandica* chronology. The previous record was characterized by a significant decline in replication prior to 1755 that precludes the use of this portion of the chronology as a statistically reliable indicator of past marine climate [37]. While numerous shells were crossdated into the earliest portion of the *A. islandica* chronology, the interval from 1729 to 1748 remains poorly replicated (just three specimens). Given the number of shells processed, the absence of identified *A. islandica* shells from this period could be interpreted as representing a potential decline in the Fladen Ground *A. islandica* population over this interval. However, the available data preclude a direct assessment of such a hypothesis. For example, shells were collected from the same vicinity as the previous [37,34] studies. Sampling was conducted over a small area of the seafloor, with eight deployments of the 1 m wide dredge at station F, and 21 deployments at station B. Therefore, subtle shifts in the distribution of *A. islandica* across these localities and the wider Fladen Ground could appear at our sampling locations as a decline in the *A. islandica* population. An alternative hypothesis could be that the *A. islandica* that lived during this interval were smaller in size than at other periods. The potentially colder conditions during this period, which coincide with the peak of the Little Ice Age, coincide with a period of reduced annual growth rates (figure 4) resulting in smaller overall shells. These potentially smaller shells would have a reduced chance of meeting the shell size thresholds used to select shells for processing. The quantitative assessment of these hypotheses requires a field campaign utilizing quantifiable sampling techniques (e.g. day grabs) and the processing of a significant number of shells across a broader array of shell sizes. Such a conjecture would provide interesting insights into the coupling of species distribution, growth rates and population dynamics with changing hydrographic and climate conditions.

While the crossdating does highlight potential challenges in the availability of *A. islandica* from some time periods, the crossdating and AAG rangefinder dating do highlight that *A. islandica* are present at these localities over the early centuries of the last millennium and beyond [41]. Ultimately, crossdating identified sufficient numbers of shells that crossdated into the chronology to attain an EPS over the 0.85 threshold for this earlier interval (1410−1650). Therefore, the potential decline or shift in the distribution of the Fladen Ground *A. islandica* population, if real, was only persistent for *ca* 100 years within the reconstructed period.

Despite the spatiotemporal distribution of *A. islandica* across the Fladen Ground being patchy, the improved replication and increased chronology EPS from 1410 to 1650 suggest that the FG RCS chronology can be used to evaluate changes in the Fladen Ground *A. islandica* population growth dating back to the early 1400s. The results of the AAG rangefinder dating, which identified numerous shells that predate the FG RCS chronology, suggest that there is significant potential to extend the chronology further [41]. It is feasible to facilitate, through additional crossdating of the AAG-dated shell material and previously undated samples, the generation of millennial length sclerochronologies from the North Sea region.

The application of the RCS detrending technique has facilitated the preservation of long-term variability in the FG RCS chronology (figures 4 and 5). Wavelet and spectral analyses highlight that the FG RCS chronology contains significant variability at approximately 200 year periodicity. This result would suggest that the application of the RCS technique has been successful in mitigating the influence of the notorious segment length curse [59]. The segment length curse is reported to typically constrain the lowest frequency variability preserved by a chronology to be equivalent to one third of the mean segment length [59]. Given the mean longevity of the shells contained in the chronology (120 ± 36 years), one might expect the lowest frequency preserved to be in the order of 40 years. Comparison of the FG RCS chronology with the herring recruitment reconstruction highlights that the two series exhibit significant covariability (*R* = 0.29, *p* < 0.05; figure 4). Visual comparison of the data also highlights that the datasets show similar patterns in the low-frequency domain. Although the herring recruitment reconstruction has been detrended over the 20th century to remove the influence of the marine Suess effect [37], the remaining portion of the record has no detrending applied and should, therefore, represent variability in herring recruitment and productivity across all frequency domains. The coherence between the δ^13^C-based record and the FG RCS chronology, therefore, suggests that the FG RCS chronology is likely capturing environmental variability across the full spectrum of frequency domains.

Comparison of the FG RCS chronology with instrumental data highlights that the record significantly covaries with a suite of environmental variables both at the sampling locations and further afield (figures 6 and 7). Previous studies have shown that *A. islandica* growth exhibits generally weak correlations with sea surface temperature (e.g. [17,18,34,60,61]). Our results support this conclusion, with no significant correlations being identified between the FG RCS chronology and sea surface temperatures at the sampling location. However, the results of the point correlation analyses and linear regression analyses, tested with the EMC method, suggest that the correlation with sea surface temperature likely does not reflect the true relationship between *A. islandica* growth and seawater temperature. The identification of significant correlations between the FG RCS chronology and mean summer seawater temperature at 35 m highlights that subsurface processes should be considered to fully characterize the nature of *A. islandica* climate–growth relationships. We hypothesize that the significant correlation between the FG RCS chronology and mean summer seawater temperature at 35 m depth is likely an indirect relationship brought about by links to mixing, stratification and ultimately the supply of food from the surface layer to the seafloor.

Observational studies have highlighted that the mixed layer depth in the Fladen Ground is highly variable during periods of stratification on daily to monthly time scales [62]. The depth of the identified correlations between the FG RCS and seawater temperature (35 m) is proximal to the observed base of the mixed layer in the northern North Sea (30−50 m [62,63]). Weston *et al*. [63] identified that there is near continuous productivity during the summer at the base of the mixed layer brought about by the mixing of nutrient-rich deep waters into the photic zone. This deep chlorophyll maxima, which occurs around 30 m depth, can account for 58% of the total water column productivity in the North Sea [63] and has been strongly linked to driving broader pelagic productivity [64]. Productivity in deep chlorophyll maxima is influenced by the mixing of the deep nutrient-rich layer into the surface layer. Increases in the rate of mixing of these layers is associated with changes in wind stress and SLP. Point correlation analyses highlight the strong link between seawater temperatures at 35 m at the sampling location and wind stress and SLP (electronic supplementary material, figure S7). This result fits with previous studies that have highlighted the strong links between the quantity and quality of food and *A. islandica* growth [23,25,38,39]. The link between the deep chlorophyll maxima and pelagic productivity is also reflected in the significant correlation identified between the FG RCS chronology and herring recruitment. However, as Weston *et al*. [63] highlight, productivity in the deep chlorophyll maxima is not observed in most observational datasets that focus on surface productivity. This could explain the lack of direct correlation between the FG RCS chronology and summer productivity in the CPR dataset. In addition, the flux of biological material to the seafloor (i.e. seston and marine snow) is also not widely captured in observational datasets [65,66]. Further work is therefore needed to better characterize the role of subsurface primary production in driving *A. islandica* growth.

The comparison of the FG RCS chronology with the CPR primary productivity dataset does highlight significant positive correlations with mean surface productivity in January, October and winter (electronic supplementary material, figure S6 and table S4). The correlation with October productivity aligns with the autumn bloom and the breakdown of stratification/increased mixing across the region [67]. An increase in productivity in October would not have to contend with the thermocline that would otherwise constrain the transport of food to the seafloor. Fluctuations in the availability of food at this time of year, which ordinarily coincides with the end of the *A. islandica*'s mean growing season in the North Sea [26], could either extend the mean growing season or lead to an increased growth rate over the final few weeks of the growth season. The correlations with primary production in January and winter are more difficult to explain, given that winter primary production is relatively low. One hypothesis is that an earlier spring bloom may fall within winter months (December–February). Given the mixed water column at this time of year, a relative increase in phytoplankton production could lead to either an early onset of the growing season or, if growth has already started, a relative increase in the early season growth rate.

Previous studies have highlighted the key role of zooplankton in modulating the quality and quantity of food that reaches *A. islandica* [23,25,38,39]. Given that zooplankton were not included in the analyses, it is likely these data do not fully represent the true linkage between primary production and *A. islandica* shell growth at this location. More work is, therefore, needed to fully characterize this relationship.

The point correlation and linear regression analyses highlight a significant negative correlation between mean winter and spring sea surface salinity (at the sampling location) and the FG RCS chronology. This relationship implies that years with fresher surface conditions during the winter and spring coincide with enhanced shell growth. Previous studies suggest that an increase in riverine input into the North Sea can bring about both reduced salinity and increased nutrient flux [68]. This therefore brings with it a boost in productivity [6] that could lead to enhanced shell growth. This mechanism requires a coupled link between Fladen Ground hydrographic variability and wider climatic processes.

Previous studies have highlighted that sclerochronologies from the northwest European shelf seas contain significant covariability with regional Atlantic Ocean climate [17–20,69–71]. The comparison of the FG RCS chronology with the EN4 seawater temperature and salinity data, using point correlation analysis, highlights significant correlations between the FG RCS chronology and regional seawater temperatures and salinity in the northeast Atlantic Ocean (figures 6 and 7). The spatial fingerprint of the temperature point correlations is similar to the sea surface temperature-based fingerprints associated with North Atlantic circulation dynamics with positive correlations following an arc pattern from the Atlantic waters west of the British Isles and Ireland across to the south Iceland [72]. Similar, albeit stronger, patterns have been found in other sclerochronological records from the northwest European shelf seas [17,20,69,73,74]. These results would support the hypothesis that *A. islandica* records from the Fladen Ground should be sensitive to fluctuations in the strength of the Atlantic inflows into the North Sea.

The point correlation analyses also fingerprint that summertime atmospheric patterns significantly covary with the FG RCS chronology. The significant negative correlations identified with mean summer SLP and the bipolar correlations with zonal and meridional wind stress highlight that the shells exhibit enhanced growth during periods with stronger south-westerly winds over the southern UK and reduced westerlies to easterlies over the northern UK (figure 8). This configuration can lead to enhanced precipitation over the UK and northwest Europe and reduce/prevent the build-up of atmospheric blocking patterns over this region. These results align closely with modelling studies that demonstrate that changes in buoyancy and wind forcing over the northeast Atlantic influence the strength of the European Slope Current and the associated Atlantic inflows into the northern North Sea [8]. Under this paradigm, changes in the strength of atmospheric forcing (wind stress) brought about by changes in SLP gradients influence the influx of nutrient-rich Atlantic water through the Atlantic inflows into the North Sea. These same atmospheric forcings influence the strength of stratification, subsurface seawater temperatures and subsurface primary production across the Fladen Ground (electronic supplementary material, figure S7). However, the EN4 data from this region, especially at depth, contain significant autocorrelation (figure 7). The results of the EMC analyses suggest that the autocorrelation may lead to the overestimation of the significance of the correlations identified in the point correlation analysis. Therefore, more work is needed to fully evaluate the linkages between the North Atlantic Ocean and atmospheric circulation patterns and marine dynamics in the Fladen Ground and wider northern North Sea [75]. One such approach would be to utilize the growing network of available annually resolved records from across the wider northeast Atlantic and European continental shelf seas [76].

## 5. Conclusion

This study highlights the potential for establishing statistically robust sclerochronologies that can provide baselines of past marine dynamics in the North Sea over at least the last six centuries. The rangefinder AAG-dated material provides evidence that, given sufficient time, it could be possible to extend these chronologies over the entirety of the last millennium and beyond. Comparison of the FG RCS chronology and sea surface temperatures supports prior studies that indicate that *A. islandica* growth is poorly correlated with sea surface temperatures. However, our analyses suggest that this result likely does not truly represent the nature of the links between *A. islandica* growth and marine climate. The significant correlation with seawater temperature at 35 m highlights the need to consider sub-surface processes that may moderate the quantity and quality of food that reaches the *A. islandica* on the seabed. However, the lack of long-term deep chlorophyll maxima data constrains our ability to test this hypothesis. These analyses ultimately suggest that Fladen Ground *A. islandica* shell growth is likely sensitive to coupled ocean–atmosphere variability over the North Sea and adjacent northeast Atlantic region. The associated coupling of the top-down atmospheric and bottom-up Atlantic Ocean forcing on sub-surface marine dynamics in the North Sea, and associated *A. islandica* growth, are complex and need further investigation. Ultimately, exploiting the growing number of available annually resolved and absolutely dated records from across the North Sea and wider northeast Atlantic region may facilitate a quantitative understanding of the complex mechanisms that are driving contrasting ecological responses across this region [6].

## Data Availability

All code and growth ring width data analysed in this manuscript are available via Zenodo [77]. The RingMeasurer app source code is available via Zenodo [78]. The physical climate data utilized in this study are openly available via the KNMI website (https://climexp.knmi.nl/start.cgi) and the UK Met Office (https://www.metoffice.gov.uk/). The Continuous Plankton Recorder Data are available from the Marine Biological Association of the UK (https://www.cprsurvey.org/). Supplementary material is available online [79].
